# Migraine Strikes as Neuronal Excitability Reaches a Tipping Point

**DOI:** 10.1371/journal.pone.0072514

**Published:** 2013-08-29

**Authors:** Marten Scheffer, Albert van den Berg, Michel D. Ferrari

**Affiliations:** 1 Department of Aquatic Ecology & Water Quality Management, Wageningen University, Wageningen, the Netherlands; 2 MESA+ Institute for Nanotechnology, University of Twente, Enschede, the Netherlands; 3 Department of Neurology, Leiden University Medical Centre, Leiden, the Netherlands; University of Würzburg, Germany

## Abstract

Self-propagating waves of cerebral neuronal firing, known as spreading depolarisations, are believed to be at the roots of migraine attacks. We propose that the start of spreading depolarisations corresponds to a critical transition that occurs when dynamic brain networks approach a tipping point. We show that this hypothesis is consistent with current pathogenetic insights and observed dynamics. Our view implies that migraine strikes when modulating factors further raise the neuronal excitability in genetically predisposed subjects to a level where even minor perturbations can trigger spreading depolarisations. A corollary is that recently discovered generic early warning indicators for critical transitions may be used to predict the onset of migraine attacks even before patients are clinically aware. This opens up new avenues for dissecting the mechanisms for the onset of migraine attacks and for identifying novel prophylactic treatment targets for the prevention of attacks.

## Introduction

About 12% of humanity is plagued by recurring migraine attacks that are characterized by disabling headaches and associated autonomic symptoms [1]. In up to one third of patients, attacks may be associated with neurological (usually visual) aura symptoms, which are caused by the electrophysiological phenomenon of “cortical spreading depression” [2]. This is a pattern of autonomous neuronal firing activity that starts at a focal point within the brain, and subsequently propagates as a slow wave in all directions across the cortex and into subcortical brain regions, until it hits upon less sensitive regions of the brain [2], [3]. Although convincing evidence in humans is still lacking, there is increasing evidence from animal experiments that spreading depression not only is the underlying mechanism for migraine aura but also may activate the trigeminovascular “headache generating” system and thus may trigger the whole attack in at least a proportion of patients[4]–[7].

While much research has focused on the electrophysiological basis of the spreading process across the cortex [8], surprisingly little is known about the mechanisms causing spreading depression to start. When, why and how does such an outbreak of spontaneous firing strike? Several factors have been shown to play a role, ranging from genetic variants[3], [9]–[12] to hormonal fluctuations [13], [14], all leading to neuronal hyperexcitability [12], [15]. The hypothesis we put forward is that increasing excitability bring the brain to a tipping point for spreading depression, and that as this tipping point is approached, resilience of the brain decreases in the sense that increasingly small perturbations are sufficient to trigger the onset.

## Results

**Figure 1:**
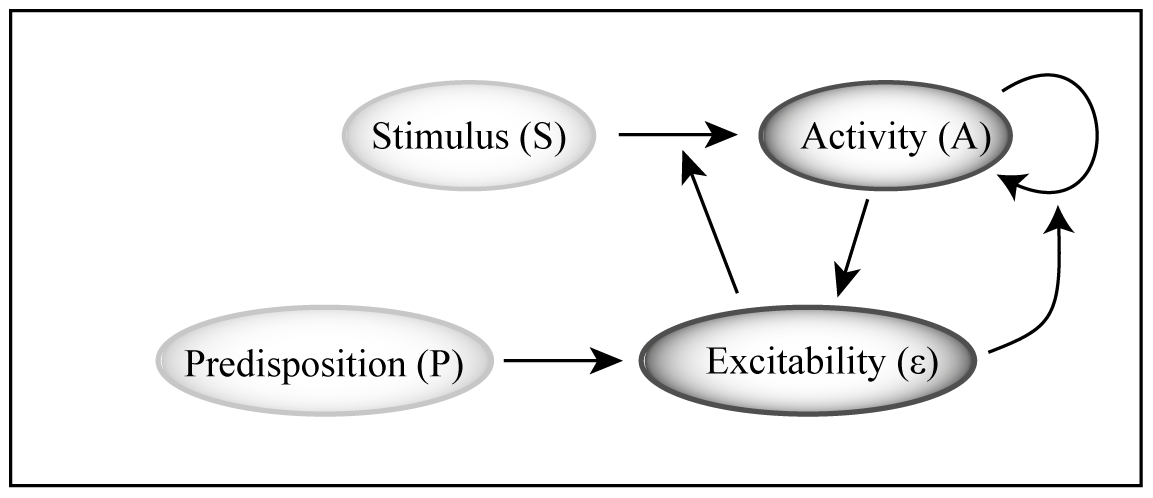
Causal structure that may lead to a tipping point for autonomous firing, as illustrated by the minimal model.

We illustrate the rationale behind our hypothesis with a minimal model of the mechanism we have in mind. The model is ‘minimal’ in the sense that we only focus on the ingredients that are essential for understanding the potential for a tipping point. There is a large literature starting in the 1970’s linking mathematics of dynamical systems to diseases with complex and cyclic dynamics such as Cheyne Stokes respiration, epilepsy, bipolar disorder, and cardiac arrhythmias [16]. Also, there have been sophisticated models in computational neuroscience that specifically address spreading depression, its onset and spatial dynamics [8], [17]. Here we do not go into such complex temporal and spatial dynamics. Rather, we focus on the simplest set of mechanisms needed to understand how the brain can arrive at a tipping point for spreading depression. We do not do explicitly consider the dynamics of individual neurons and their interactions in the complex network. Instead, we analyze the total activity and excitability of a local ‘population of neurons’ (a so-called mean field- or mass- model). This allows us to explain the essence based on only three assumptions (Fig. 1):

1. Activity of a group of neurons (A) increases with the level of incoming stimuli (S) and the overall neuronal excitability (ε).
2. Excitability is modulated by overall physiological conditions and genetic factors, and promoted by local neural activity, implying positive feedback mechanisms.
3. When excitability surpasses a critical level, neural activity may be triggered merely by the activity of neighboring neurons, thus turning into a locally contagious process.

While the first assumption is straightforward, the background for the second assumption is that neural activity promotes intercellular concentrations of potassium and glutamate, which in turn promote the excitability of neurons in the area [18]. The third assumption follows from the observation that cortical spreading depression spreads centrifugally as a traveling wave over the cortex. Unless orchestrated from another brain area (for which there is no indication) this behavior implies a local contagious component to the firing of neurons.

**Figure 2:**
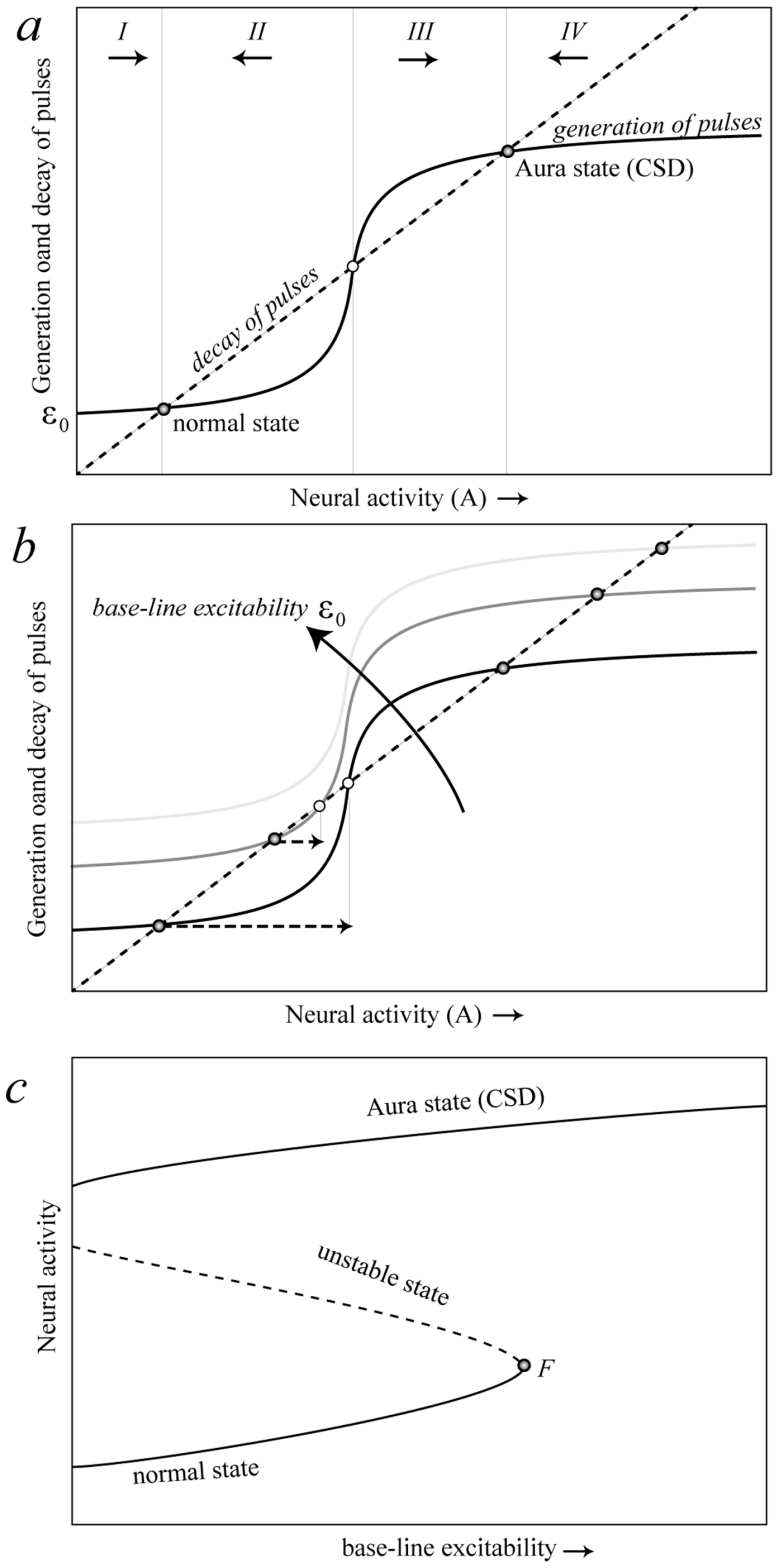
Graphical model showing how a tipping point for cortical spreading depression can arise. a) Three equilibriums may occur at intersection points where the rate of generation of new pulses (sigmoidal curve) equals the rate of decay (dashed line) of neural pulses. Activity increases when the generation of new pulses exceeds the decay of pulses (sections I and III) and decreases in the other sections (sections II and IV). It can be seen from the arrows representing this direction of change that the middle intersection point is a repellor that marks the border between the basins of attraction of the two alternative stable states. b) Increasing base-line excitability promotes the generation of new pulses causing the unstable equilibrium (open dot) and the stable normal state (left hand solid dot) to move closer together. This reduces resilience of the normal state in the sense that a smaller perturbation is needed to invoke a shift to the Aura state (horizontal dashed arrows in panel). c) Plotting how the intersection points representing equilibriums move as a function of base-line excitability, a catastrophe fold arises. The fold bifurcation point (F) marks the loss of stability of the normal state.

Figure 2 explains graphically how a tipping point can arise from the above assumptions (for the mathematical model, and a more specific definition of the parameters and variables see the *material and methods* section). Imagine a dynamic equilibrium of neural activity in a small brain region, resulting from the balance between generation and decay of pulses. If we plot the generation and decay of pulses together, it can be seen that multiple equilibriums may arise at their intersections if the generation of activity rises relatively sharply when the contagious local neural firing kicks in (positive feedback) around a critical excitability level (*ε_crit_*). The unstable middle equilibrium marks the boundary between the two ‘basins of attraction’ around the stable states.

**Figure 3:**
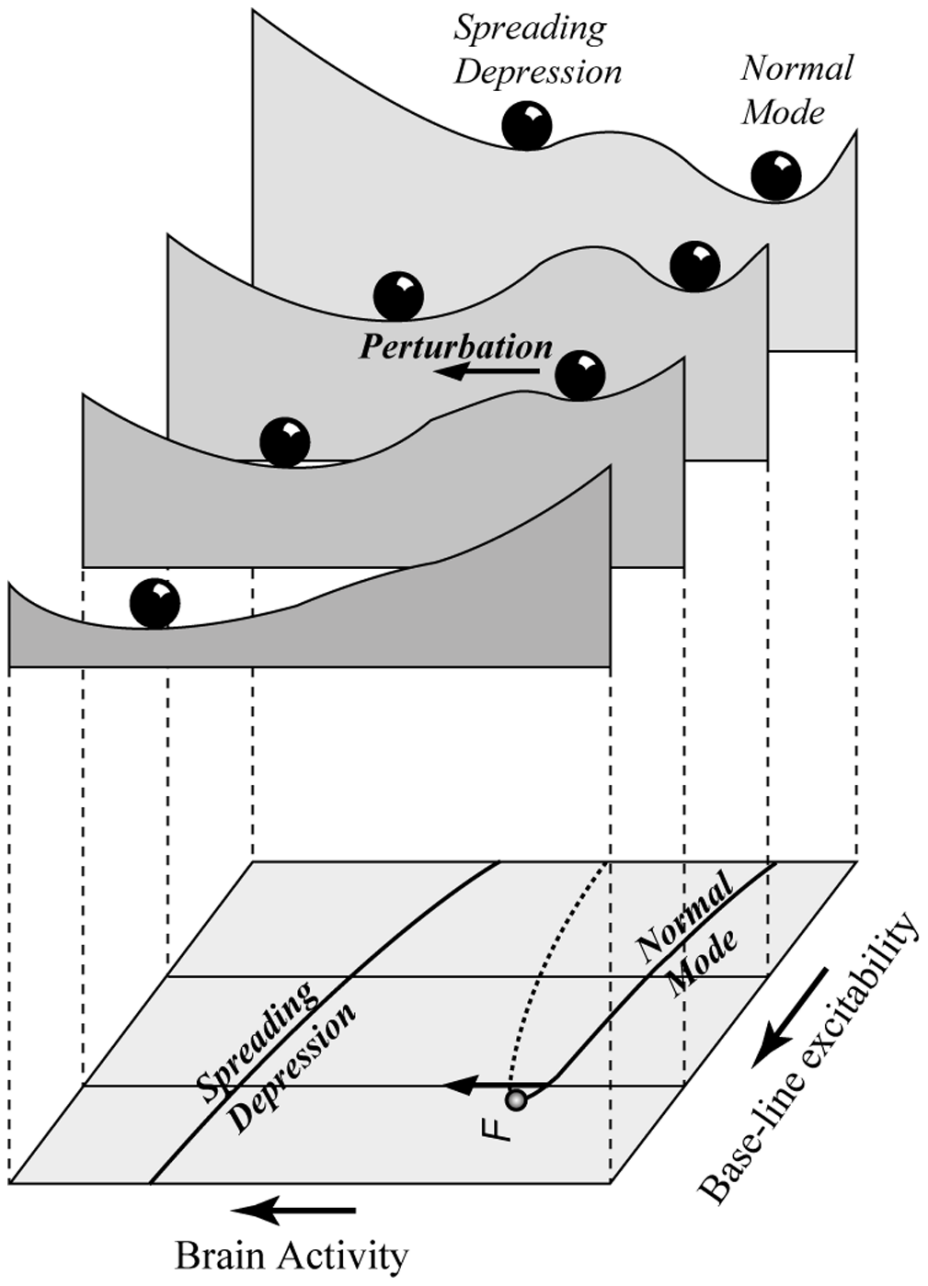
Stability landscape interpretation of how resilience of the normal mode of brain activity can be lost at high levels of base-line excitability as determined by genetically coded or other physiological conditions. The catastrophe fold at the base plane corresponds to the one depicted in figure 2c.

To see how the brain can be brought to a tipping point, imagine what happens if the base-line excitability *ε_0_* increases (Fig. 2b). The unstable point moves closer to the normal stable state causing the basin of attraction for the normal state to shrink. Therefore the resilience becomes smaller, in the sense that a smaller perturbation is needed to let the system fall into the attraction basin of the hyperactive (aura/migraine) state. A further increase in base-line excitability can cause the unstable point to collide with the normal stable state, causing it to lose stability and disappear. This is the real ‘tipping point’, or in mathematical terms a fold-bifurcation point. The way in which the equilibriums are affected by base-line excitability (and/or stimulus strength) can be summarized in one graph (Fig. 2c) known as a catastrophe fold, where the dashed middle section represents the repelling unstable equilibriums that mark the border of the attraction basins of the normal state and the aura state. The essence of the interpretation of changing resilience can be captured in an intuitive way by a stability landscape representation (fig. 3). Each of the landscapes corresponds to a different physiological condition (captured in excitability *ε_0_* in our minimal model). Note that the aura/migraine state in practice is a transient rather than a true stable state. Hyperactivity stops at the rear end of the travelling wave of cortical spreading depression as a result of exhaustion of ATP or oxygen. We are not further addressing the mechanisms of how the process spreads and ultimately halts [17], as we focus on the triggering mechanisms.

## Discussion

The presence of a tipping point implies four well-defined predictions that can be checked against empirical observations [19], [20]:

1. The system occasionally goes through a sudden transition.
2. A system at any given moment tends to be in either of the two contrasting states rather than somewhere in between.
3. A short perturbation can trigger a shift to a lasting contrasting regime.
4. Change in conditions can reduce the resilience of the system in the sense that a smaller perturbation is needed to invoke a shift.

Clearly the character of cortical spreading depression is well in line with these predictions: The onset of cortical spreading depression sharply breaks the normal regime of the brain (1); It is an all-or-none phenomenon with no evidence for stable intermediate states (2) and; Perturbations such as light flashes can invoke a shift (3) [18]. The fact that the latter happens only rarely in non-migraineurs and that an attack may sometimes be provoked by even a small stimulus (e.g. a light flash) implies indirect evidence for prediction (4). In addition, the observation that the amplitude of electroencephalographic responses to light flashes tends to be systematically larger in the days before a migraine attack [21], [22] is well in line with the view that increased excitability may periodically bring patients closer to a tipping point.

The view of cortical spreading depression-induced migraine as a critical transition at a tipping point opens up entirely new angles when it comes to perspectives for attack prediction and treatment. Most importantly, it has recently been shown that the distance to a tipping point in complex systems may be estimated based on generic early warning signals [19]. For instance, the recovery time to the normal regime upon a small perturbation is predicted to become longer as a system comes closer to the critical point [19], [23]. This has just been demonstrated for a living system [24], and it would be a logical step to study the decay rate of brain responses to small perturbations such as visual stimuli. As the brain continuously generates activity, development of a robust protocol to detect such slowing down is not trivial. However, if we find ways to objectively measure resilience this way, this opens up novel possibilities. Firstly, we may give patients a simple home test to warn for impending attacks. They could then start pre-emptive short term prophylaxis to reduce excitability and enhance resilience, e.g with antiepileptic agents known to be also effective in migraine [25], and in addition avoid strong stimuli or activities which are known to specifically trigger their migraine attacks. Secondly, from a research perspective, objective measures of resilience would help to effectively search for mechanisms involved. Now we typically classify subjects into groups such as migraineurs versus healthy persons. However, in our view resilience within these groups may vary strongly, and also within subjects resilience will vary over time. Objective measurements of resilience would greatly enhance the power for instance of statistical tools that can put us on the track of factors related to resilience, including time-varying aspects such as blood or cerebrospinal levels of substances. Some factors contributing to resilience are now well understood, but much could be gained in terms of treatment design if we could develop a more comprehensive insight in the mechanisms that can strengthen or undermine resilience against the onset of cortical spreading depression.

Finally, other episodic brain disorders such as epilepsy may benefit as well from this type of research. Migraine and epilepsy are comorbid episodic brain disorders that have common pathophysiologic mechanisms and treatments. Migraine attacks, like epileptic seizures, may be triggered by excessive neocortical cellular excitability; in migraine, however, the hyperexcitability is believed to transition to cortical spreading depression rather than to the hyper-synchronous activity that characterizes seizures [25].

## Materials and Methods

To translate our three assumptions into a mathematical model, imagine a dynamic equilibrium of neural activity (*A*) in a small region, resulting from the generation and decay of pulses. A simple way to formulate that consistent with our assumptions could be:
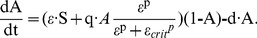
(1)


The last term in this equation reflects a simple proportional decay (at rate *d*) of pulses. The rest of the equation describes the generation of pulses. The multiplier (1- *A*) goes to zero as *A* approaches unity, causing activity to be limited to values between zero and one. The term *ε·S* represents the generation of pulses resulting from external stimuli (*S*) depending on the excitability (*ε*) of the neurons. The remaining term reflects the generation of pulses caused by the firing activity (*A*) of the other neurons in the area. The maximum intensity of this ‘contagion effect’ is scaled by a factor (*q*). The effect really kicks in when a critical excitability (*ε_crit_*) is reached. This threshold response is formulated as the so-called Hill function (*ε^p^/*(*ε^p^*+*ε_crit_^p^*)) that for sufficiently high values of *p* increases with *ε* in a steep sigmoidal way from zero to one around the critical excitability level (*ε_crit_*).

To include the feedback effect of activity on excitability we assume a linear relationship for simplicity:
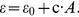
(2)


Where *ε_0_* is the base-line excitability as determined by predisposition through genetic factors and general physiological conditions, and *c* is the steepness of increase in excitability with neural activity. The full model is thus defined by substituting equation 2 for each *ε* in equation 1:
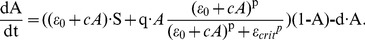
(3)


For suitable parameter settings (e.g. our defaults: S = 0.1; d = 0.1; e = 1.5; f = 1; h = 5; p = 4) this model has a tipping point where the system may switch between a normal activity regime to a state of self-propelled hyperactivity (the spreading depression). To illustrate in an intuitive way why such behavior can arise from our assumptions we turn to a simple graphical representation.

## References

[1] Goadsby PJ, Lipton RB, Ferrari MD (2002) Migraine - Current understanding and treatment. New England Journal of Medicine 346: 257–270. 11807151 10.1056/NEJMra010917

[2] Lauritzen M (1994) Pathophysiology of the migraine aura: The spreading depression theory. Brain 117: 199–210. 7908596 10.1093/brain/117.1.199

[3] Eikermann-Haerter K, Yuzawa I, Qin T, Wang Y, Baek K, et al (2011) Enhanced subcortical spreading depression in familial hemiplegic migraine type 1 mutant mice. Journal of Neuroscience 31: 5755–5763. 21490217 10.1523/JNEUROSCI.5346-10.2011 PMC3135337

[4] Dalkara T, Zervas NT, Moskowitz MA (2006) From spreading depression to the trigeminovascular system. Neurological Sciences 27: s86–s90. 16688636 10.1007/s10072-006-0577-z

[5] Zhang X, Levy D, Kainz V, Noseda R, Jakubowski M, et al (2011) Activation of central trigeminovascular neurons by cortical spreading depression. Annals of Neurology 69: 855–865. 21416489 10.1002/ana.22329 PMC3174689

[6] Levy D, Moskowitz MA, Noseda R, Burstein R (2012) Activation of the migraine pain pathway by cortical spreading depression: Do we need more evidence? Cephalalgia 32: 581–582. 21996564 10.1177/0333102411424621 PMC3698973

[7] Karatas H, Erdener SE, Gursoy-Ozdemir Y, Lule S, Eren-Kocak E, et al (2013) Spreading Depression Triggers Headache by Activating Neuronal Panx1 Channels. Science 339: 1092–1095. 23449592 10.1126/science.1231897

[8] Dahlem MA, Hadjikhani N (2009) Migraine aura: Retracting particle-like waves in weakly susceptible cortex. PLoS ONE 4. 10.1371/journal.pone.0005007 PMC2659426 19337363

[9] Van Den Maagdenberg AMJM, Pizzorusso T, Kaja S, Terpolilli N, Shapovalova M, et al (2010) High cortical spreading depression susceptibility and migraine-associated symptoms in Cav2.1 S218L mice. Annals of Neurology 67: 85–98. 20186955 10.1002/ana.21815

[10] Eikermann-Haerter K, Moskowitz MA (2008) Animal models of migraine headache and aura. Current Opinion in Neurology 21: 294–300. 18451713 10.1097/WCO.0b013e3282fc25de

[11] Ayata C, Jin H, Kudo C, Dalkara T, Moskowitz MA (2006) Suppression of cortical spreading depression in migraine prophylaxis. Annals of Neurology 59: 652–661. 16450381 10.1002/ana.20778

[12] Tottene A, Conti R, Fabbro A, Vecchia D, Shapovalova M, et al (2009) Enhanced Excitatory Transmission at Cortical Synapses as the Basis for Facilitated Spreading Depression in Ca_V_2.1 Knockin Migraine Mice. Neuron 61: 762–773. 19285472 10.1016/j.neuron.2009.01.027

[13] Eikermann-Haerter K, Baum MJ, Ferrari MD, Van Den Maagdenberg AMJM, Moskowitz MA, et al (2009) Androgenic suppression of spreading depression in familial hemiplegic migraine type 1 mutant mice. Annals of Neurology 66: 564–568. 19847904 10.1002/ana.21779 PMC2783310

[14] Eikermann-Haerter K, Dileköz E, Kudo C, Savitz SI, Waeber C, et al (2009) Genetic and hormonal factors modulate spreading depression and transient hemiparesis in mouse models of familial hemiplegic migraine type 1. Journal of Clinical Investigation 119: 99–109. 19104150 10.1172/JCI36059 PMC2613474

[15] Moskowitz MA, Bolay H, Dalkara T (2004) Deciphering Migraine Mechanisms: Clues from Familial Hemiplegic Migraine Genotypes. Annals of Neurology 55: 276–280. 14755732 10.1002/ana.20035

[16] Glass L, Mackey MC (1988) From clocks to chaos: the rhythms of life. Princeton: Princeton University Press. 248 p.

[17] Dahlem MA, Graf R, Strong AJ, Dreier JP, Dahlem YA, et al.. (2010) Two-dimensional wave patterns of spreading depolarization: Retracting, re-entrant, and stationary waves. Physica D: Nonlinear Phenomena in press.

[18] Bowyer SM, Aurora SK, Moran JE, Tepley N, Welch KMA (2001) Magnetoencephalographic fields from patients with spontaneous and induced migraine aura. Annals of Neurology 50: 582–587. 11706963 10.1002/ana.1293

[19] Scheffer M, Bascompte J, Brock WA, Brovkin V, Carpenter SR, et al (2009) Early-warning signals for critical transitions. Nature 461: 53–59. 19727193 10.1038/nature08227

[20] Scheffer M, Carpenter SR (2003) Catastrophic regime shifts in ecosystems: linking theory to observation. Trends in Ecology & Evolution 18: 648–656.

[21] Kropp P, Gerber WD (1993) Is increased amplitude of contingent negative varation in migraine due to cortical hyperactivity or to reduced habituation? Cephalalgia 13: 37–41. 8448787 10.1046/j.1468-2982.1993.1301037.x

[22] Shibata K, Yamane K, Otuka K, Iwata M (2008) Abnormal visual processing in migraine with aura: A study of steady-state visual evoked potentials. Journal of the Neurological Sciences 271: 119–126. 18495160 10.1016/j.jns.2008.04.004

[23] Van Nes EH, Scheffer M (2007) Slow recovery from perturbations as a generic indicator of a nearby catastrophic shift. American Naturalist 169: 738–747. 10.1086/516845 17479460

[24] Veraart AJ, Faassen EJ, Dakos V, van Nes EH, Lürling M, et al (2012) Recovery rates reflect distance to a tipping point in a living system. Nature 481: 357–359. 10.1038/nature10723 22198671

[25] Rogawski MA (2008) Common pathophysiologic mechanisms in migraine and epilepsy. Archives of Neurology 65: 709–714. 18541791 10.1001/archneur.65.6.709

